# Prognostic value of alveolar volume in systolic heart failure: a prospective observational study

**DOI:** 10.1186/1471-2466-13-69

**Published:** 2013-11-22

**Authors:** Massimo Miniati, Simonetta Monti, Matteo Bottai, Ivana Pavlickova, Claudio Passino, Michele Emdin, Roberta Poletti

**Affiliations:** 1 Dipartimento di Medicina Sperimentale e Clinica, Università di Firenze, Viale Morgagni 85, 50134 Firenze, Italy; 2 Istituto di Fisiologia Clinica del CNR, Pisa, Italy; 3 Fondazione CNR-Regione Toscana "G. Monasterio", Pisa, Italy; 4 Unit of Biostatistics, Karolinska Institutet, Stockholm, Sweden; 5 Division of Biostatistics, Arnold School of Public Health, University of South Carolina, Columbia, SC, USA; 6 Scuola Superiore di Studi Universitari e Perfezionamento "S. Anna", Pisa, Italy

**Keywords:** Systolic heart failure, Alveolar volume, Prognosis, Survival

## Abstract

**Background:**

Ventilatory impairment is known to occur in patients with heart failure (HF). Alveolar volume (V_A_) is measured by the dilution of an inert gas during a single breath-hold maneuver. Such measurement is sensitive to ventilatory disturbances. We conducted a prospective, observational study to establish the prognostic value of V_A_ in systolic HF.

**Methods:**

We studied 260 consecutive patients who were hospitalized for systolic HF. All patients were evaluated under stable clinical conditions, before hospital discharge. Lung function studies included spirometry and determination of the lung diffusing capacity for carbon monoxide (DL_CO_) by the single-breath method. We also measured the cardiothoracic ratio on frontal chest radiographs, and the circulating levels of N-terminal pro-hormone of B-type natriuretic peptide (NT-proBNP). The hazard ratio (HR) of death was estimated with Cox regression, and the percentiles of survival time with Laplace regression. For survival analysis, V_A_ was categorized as < 80% (n = 135), or ≥ 80% of the predicted value (n = 125).

**Results:**

Follow-up had a median duration of 2.7 years (interquartile range, 1.1 to 4.2 years). The crude mortality rate was 27% in the whole sample, 36% in patients with V_A_ < 80%, and 16% in those with V_A_ ≥ 80%. The HR of death was 2.3-fold higher in patients with V_A_ < 80% than in those with V_A_ ≥80% (p = 0.002). After adjusting for age, New York Heart Association class III-IV, cardiothoracic ratio >0.5, NT-proBNP, persistent atrial fibrillation, DL_CO_, COPD comorbidity, use of beta-blockers and angiotensin converting enzyme inhibitors, the HR decreased to 1.9 but remained statistically significant (p = 0.039). Two percent of the patients with V_A_ < 80% died about 0.9 years earlier than those with V_A_ ≥ 80% (p = 0.033). The difference in survival time at the 20th percentile was 0.8 years.

**Conclusions:**

V_A_ is a significant, independent predictor of reduced survival in patients with systolic HF.

## Background

Heart failure (HF) is a common clinical disorder carrying significant morbidity and mortality [1,2]. Ventilatory impairment is known to occur in patients with HF, and is ascribed to factors such as heart enlargement, pulmonary stiffness due to chronic interstitial edema, and respiratory muscle dysfunction [3-7]. The coexistence of chronic airflow obstruction may add to ventilatory dysfunction, and is believed to have a negative prognostic impact in patients with chronic HF [8].

As of now, most of the studies on the prognostic impact of respiratory dysfunction in HF focused on simple spirometry [9-14], with only a few exploring the exchange of gases across the alveolar-capillary membrane [15,16].

Alveolar volume (V_A_) is the volume of air in the lung available for gas exchange, and is currently measured by the dilution of an inert gas during a single breath-hold maneuver [17]. Due to the single-breath approach, such measurement is sensitive to ventilatory disturbances. In healthy subjects, V_A_ equals total lung capacity (TLC) measured by multiple-breath helium dilution. In subjects with ventilatory impairment, V_A_ is often much lower than TLC because of the insufficient mixing of gas in alveolar spaces [18].

We conducted a prospective, observational study aimed at establishing the prognostic value of V_A_, as % of the predicted value, in 260 consecutive patients with systolic HF. The outcome measure was all-cause mortality over a median follow-up time of about three years.

## Methods

### Ethics statement

The protocol was approved by the local ethics committee (Comitato Etico, Azienda Ospedaliero-Universitaria Pisana, Pisa, Italy). Before entering the study, the subjects provided an informed written consent.

### Sample

The study sample comprised 260 consecutive patients with an established diagnosis of systolic HF. They were hospitalized at the CNR Institute of Clinical Physiology and "G. Monasterio" Foundation, Pisa, Italy, between January 1, 2000 and December 31, 2007. The diagnosis of HF was based on the Framingham criteria [19], and the finding of a left ventricular ejection fraction (LVEF) < 50% at transthoracic echocardiography. Patients were excluded if they had active cancer, recent (within 6 months) acute coronary syndromes, or pulmonary arterial hypertension.

### Study protocol

All the patients were evaluated under stable clinical conditions, shortly before hospital discharge. Lung function studies included the measurement of slow (SVC) and forced vital capacity (FVC), forced expiratory volume in one second (FEV_1_), and total lung capacity (TLC). At least three spirometric measurements were obtained and the highest values were chosen. Spirometry was performed by experienced technologists in conformity with the ATS/ERS standards [20]. The diagnosis of COPD was based on clinical and spirometric criteria (post-bronchodilator ratio of FEV_1_/SVC below the 5th percentile of predicted value) [21]. The degree of airflow obstruction was categorized as mild, moderate, or severe if FEV_1_ was > 80%, between 80 and 50%, and < 50% of predicted, respectively. Ventilatory restriction was diagnosed if FEV_1_/SVC ratio was normal, and TLC below the 5th percentile of predicted [21]. The diffusing capacity of the lung for carbon monoxide (DL_CO_) was determined using the single-breath method in conformity with ATS/ERS recommendations [17]. V_A_ was measured during the single-breath maneuver using helium as the inert gas [17].

Postero-anterior and lateral digital chest radiographs were obtained in all the subjects at the time of lung function testing, and were examined by two of the authors (MM, SM) for the presence of heart, pulmonary, and pleural abnormalities. On the postero-anterior view, we also measured the cardiothoracic ratio. This was regarded as abnormal if > 0.5. We measured the circulating levels of hemoglobin, creatinine, and N-terminal pro-hormone of B-type natriuretic peptide (NT-proBNP). The latter was measured by an electrochemiluminescent sandwich immonoassay using Elecsys 2010 analyser (Roche Diagnostics, Germany) [22]. Medications prescribed at the time of hospital discharge were also recorded.

### Follow-up

The 260 patients were followed up until death or March 31, 2009, whichever occurred first. All the subjects were seen periodically at the outpatient clinic of our institution. Whenever required, their family physicians were also contacted. The cause of death was established by reviewing medical records, autopsy findings, or death certificates.

### Statistical analysis

Differences between groups were assessed by Fisher’s exact test for the categorical variables, and by Mood’s median test for the continuous variables. Continuous variables in the text and in the tables are reported as median and interquartile range (IQR). The association between V_A_ and survival was evaluated with two methods: Cox proportional hazard regression to estimate hazard ratio (HR) for the risk of death, and Laplace regression to estimate the percentiles of survival time [23]. Survival percentiles may offer a comprehensive picture of the covariate effects on survival time while overcoming some of the known interpretational limitations of HR [24]. The main exposure of interest was V_A_ which was categorized as < 80% or ≥ 80% of the predicted value. Based on univariate analysis, the following potential confounders were considered: tertiles of age (≤ 61, 62-71, ≥ 72 years), New York Heart Association (NYHA) class III-IV, cardiothoracic ratio > 0.5, DL_CO_ as % predicted (split in two categories by the median value in the sample), tertiles of NT-proBNP (≤ 787, 788-2058, ≥ 2059 pg/mL), persistent atrial fibrillation, COPD comorbidity, use of beta-blockers, and of angiotensin converting enzyme (ACE) inhibitors. The crude and adjusted percentiles, and the hazard ratios and associated 95% confidence intervals (CI) are reported. The proportionality of the hazard in the Cox regression was tested based on the Schoenfeld residuals for each model separately. Two-tailed p-values < 0.05 were considered statistically significant throughout. The statistical analysis was performed with Stata version 11 (StataCorp, College Station, TX).

## Results

### Sample

The baseline characteristics of the study sample are given in Table 1. Based on clinical and spirometric data, COPD was diagnosed in 65 (25%) of 260 cases; airflow obstruction was mild in 11 patients (17%), moderate in 41 (63%), and severe in 13 (20%). In the whole sample, the median value of V_A_ was 79% of predicted (IQR, 70 to 88%). There was a highly significant, inverse correlation of V_A_ with NT-proBNP (r = -0.21; p < 0.001) and cardiothoracic ratio (r = -0.39; p < 0.001), and a weaker borderline significant correlation with LVEF (r = +0.11; p = 0.054).

**Table 1:** Baseline characteristics of 260 patients with systolic heart failure

**Characteristic**	**Number or Median**	**(Percent or IQR)**
Age, years	68	(58–75)
Male gender	207	(80)
BMI, kg/m^2^	26	(24–29)
Current smoker	50	(19)
NYHA class III-IV	84	(32)
LVEF, %	32	(25–40)
Cardiothoracic ratio >0.5	162	(62)
NT-ProBNP, pg/mL	1302	(531–3028)
Hemoglobin, g/dL	13.7	(12.4–14.9)
Creatinine, mmol/L	101	(82–127)
FEV_1_, % predicted	87	(72–101)
DL_CO_, % predicted	70	(61–81)
COPD	65	(25)
Ventilatory restriction	53	(20)
Coronary artery disease	115	(44)
Hypertension	141	(54)
Persistent atrial fibrillation	51	(20)
Prior stroke	21	(8)
Diabetes	82	(32)
Dyslipidemia	109	(42)
Beta-blockers	223	(86)
ACE-inhibitors	150	(58)
Angiotensin receptor antagonists	75	(29)
Loop diuretics	225	(87)
Potassium sparing drugs	175	(67)
Warfarin	42	(16)
Implanted pace-maker	35	(13)
Implanted defibrillator	13	(5)

IQR = interquartile range. BMI = body mass index. NYHA = New York Heart Association. LVEF = left ventricular ejection fraction. NT-proBNP = N-terminal pro-hormone of B type natriuretic peptide. FEV_1_ = forced expiratory volume in one second. DL_CO_ = diffusing capacity of the lung for carbon monoxide. COPD = chronic obstructive pulmonary disease. ACE = angiotensing converting enzyme.

Table 2 shows the characteristics of the study sample spit by V_A_ category. As compared with patients having V_A_ ≥ 80%, those with V_A_ < 80% featured the following statistically significant differences: (a) older age; (b) higher prevalence of NYHA class III-IV, abnormal cardiothoracic ratio, persistent atrial fibrillation, COPD comorbidity, and ventilatory restriction; (c) lower FEV_1_ and DL_CO_; (d) higher levels of NT-proBNP. As regards medical treatment, significantly less patients with V_A_ < 80% were prescribed beta-blockers and ACE-inhibitors, and significantly more were on oral anticoagulants than those with V_A_ ≥ 80%.

**Table 2:** Study sample split by alveolar volume

	**Alveolar volume (% predicted)**				
**Characteristic**	**<80 (n = 135)**		**≥80 (n = 125)**		**P-value**
Age, years	71	(66–77)	64	(55–71)	<0.001
Male gender	109	(81)	98	(78)	0.648
BMI, kg/m^2^	26	(24–29)	26	(23–30)	1.000
Current smoker	21	(16)	29	(23)	0.157
NYHA class III-IV	57	(42)	27	(22)	<0.001
LVEF, %	30	(25–38)	33	(25–40)	0.710
Cardiothoracic ratio >0.5	93	(69)	69	(55)	0.029
NT-ProBNP, pg/mL	1607	(811–3878)	982	(297–2141)	0.003
Hemoglobin, g/dL	13.6	(12.5–14.7)	13.7	(12.4–15.0)	0.552
Creatinine, mmol/L	105	(82–137)	97	(82–114)	0.139
FEV_1_, % predicted	78	(65–87)	99	(90–109)	<0.001
DL_CO_, % predicted	66	(53–75)	76	(68–92)	<0.001
COPD	42	(31)	23	(18)	0.022
Ventilatory restriction	53	(39)	0	(0)	<0.001
Coronary artery disease	66	(49)	49	(39)	0.134
Hypertension	75	(56)	66	(53)	0.709
Persistent atrial fibrillation	34	(25)	17	(14)	0.020
Prior stroke	10	(7)	11	(9)	0.821
Diabetes	44	(33)	38	(30)	0.790
Dyslipidemia	52	(39)	57	(44)	0.260
Beta-blockers	107	(79)	116	(93)	0.002
ACE-inhibitors	69	(51)	81	(65)	0.033
Angiotensin receptor antagonists	40	(30)	35	(28)	0.786
Loop diuretics	117	(87)	108	(86)	1.000
Potassium sparing drugs	84	(62)	91	(73)	0.085
Warfarin	28	(21)	14	(11)	0.043
Implanted pace-maker	20	(15)	15	(12)	0.589
Implanted defibrillator	5	(4)	8	(6)	0.398

Data are numbers (%), or medians (interquartile range). For abbreviations see Table 1.

Radiologic signs of interstitial lung edema were detected in 16% of the patients with V_A_ < 80% and in 3% of those with V_A_ ≥ 80% (p < 0.001). Similarly, small pleural effusions prevailed significantly in the lower V_A_ category than in the other (24% vs 6%, p < 0.001), whereas no statistically significant difference was observed as regards the prevalence of radiologic signs of emphysema (11% vs 7%, p = 0.293). None of the sampled patients had evidence of overt lung fibrosis.

### Survival analysis

Follow-up was completed in all the patients, and had a median duration of 2.7 years (IQR, 1.1 to 4.2 years). The crude mortality rate was 27% (69/260) in the whole sample, 36% (49/135) in patients with V_A_ < 80%, and 16% (20/125) in those with V_A_ ≥ 80%. The causes of death were: heart failure (n = 44), cancer (n = 9), respiratory failure (n = 5), sudden death (n = 4), liver failure (n = 2), sepsis (n = 2), multiple trauma (n = 2), and renal failure (n = 1).

**Figure 1:**
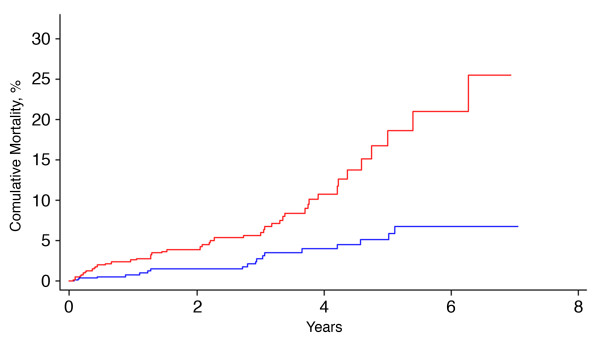
**Estimated cumulative mortality in the study sample split by alveolar volume (V**_**A**_**) as % predicted: V**_**A **_**<80% (red step curve); V**_**A **_**≥80% (blue step curve).** Estimates are adjusted for age, NYHA class III-IV, cardiothoracic ratio >0.5, NT-proBNP, persistent atrial fibrillation, DL_CO_ as % predicted, and COPD comorbidity. P-value by log-rank test = 0.032.

Figure 1 shows the estimated cumulative incidence of death which was significantly higher in patients with V_A_ < 80% than in the others (p = 0.032). The mortality curves are adjusted for age, NYHA class III-IV, cardiothoracic ratio >0.5, NT-proBNP, persistent atrial fibrillation, DL_CO_ as % predicted, and COPD comorbidity.

Table 3 describes the regression estimates of the HR of death for the two categories of V_A_. The unadjusted HR of death in patients with V_A_ < 80% was 2.3-fold higher than in those with V_A_ ≥ 80% (p = 0.002). After adjusting for potential confounders, the HR of death decreased to 1.9 but remained statistically significant (p = 0.039).

**Table 3:** Estimated hazard ratios of death by alveolar volume category

	**V**_**A **_**<80% versus V**_**A **_**≥80%**		
**Model**	**Hazard ratio**	**95% CI**	**P-value**
1	2.31	(1.37–3.90)	0.002
2	1.90	(1.03–3.50)	0.039

V_A_ = alveolar volume (as % predicted). CI = confidence interval.

Model 1: unadjusted.

Model 2: adjusted for age, NYHA class III-IV, NT-proBNP, cardiothoracic ratio >0.5, persistent atrial fibrillation, DL_CO_ as % predicted, COPD comorbidity, use of beta-blockers and of ACE-inhibitors.

For abbreviations, see Table 1.

**Figure 2:**
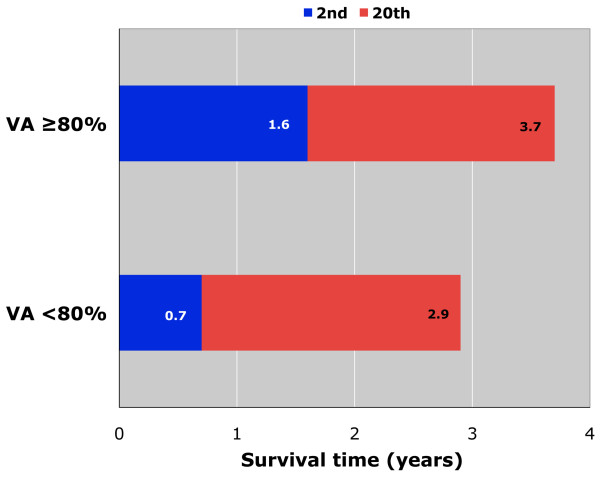
**Laplace regression estimates of the 2nd and 20th percentile of survival time in the sample split by alveolar volume (V**_**A**_**) as % predicted (<80% vs ≥80%).** Estimates are adjusted for age, NYHA class III-IV, cardiothoracic ratio >0.5, NT-proBNP, persistent atrial fibrillation, DL_CO_ as % predicted, and COPD comorbidity.

Laplace regression estimates of the percentiles of survival by V_A_ category are displayed in Figure 2. They are adjusted for age, NYHA class III-IV, cardiothoracic ratio > 0.5, NT-proBNP, persistent atrial fibrillation, DL_CO_ as % predicted, and COPD comorbidity. Two percent of the patients with V_A_ < 80% died about 0.9 years earlier than those with V_A_ ≥ 80% (p = 0.033). The difference at the 20th percentile was 0.8 years. So, the frailest were the most affected individuals by reduced V_A_.

## Discussion

The present study was designed to establish whether V_A_, as percent of the predicted value, is a prognostic indicator in systolic HF. Our results can be summarized thus: (a) the cumulative incidence of death is significantly higher in patients with V_A_ < 80% predicted than in those with V_A_ ≥ 80%; (b) the estimated HR of death is about two-fold greater among the patients in the lower V_A_ category, and remains significantly higher after adjusting for relevant confounding variables; (c) as indicated by Laplace regression, reduced V_A_ is strongly associated with early deaths.

We found a significant, inverse correlation between V_A_ and cardiothoracic ratio. This suggests that heart enlargement is a major determinant of reduced V_A_ in chronic HF. Generalized stiffness of the lung parenchyma may also contribute to diminish V_A_. Such reduced lung distensibility is due to chronic interstitial edema, and pulmonary vascular remodeling that are known to occur in long-standing HF [25].

We also observed that V_A_ is significantly correlated with the circulating levels of NT-proBNP, an established prognostic biomarker of HF [2]. After adjusting for NT-proBNP, the HR of death associated with reduced V_A_ remains statistically significant so indicating that V_A_ is an independent predictor of survival.

A number of studies investigated on simple spirometry as a potential indicator of future cardiovascular events. In the setting of the Framingham Study, Kannel et al. observed that FVC is a significant, independent predictor of cardiovascular morbidity and mortality in allegedly healthy subjects [9]. Later, Kannel et al. reported that FVC is a predictor of subsequent cardiac failure in individuals with coronary artery disease and left ventricular hypertrophy [10].

In recent years, there has been increasing interest in evaluating the prognostic impact of COPD comorbidity in patients with chronic HF. Mascarenhas et al. [11] conducted a retrospective study in 186 patients with systolic HF, and found no significant association between having COPD and all-cause mortality (HR 1.40, 95% CI 0.88 to 2.44). Only severe airflow obstruction (FEV_1_ < 50% of predicted value) appeared to be a predictor of reduced survival (HR 2.10, 95% CI 1.05 to 4.22) [11]. In 527 patients with a clinical diagnosis of HF, Iversen et al. reported that FEV_1_ has independent prognostic value (HR 0.86 per 10% change with respect to the predicted value, p < 0.001) [12]. By contrast, Macchia et al. estimated an adjusted HR of death of 0.77 (95% CI, 0.37 to 1.58) in patients with systolic HF and concomitant chronic airway obstruction, suggesting that COPD has no significant bearing on survival [13]. The three studies differ substantially for proportion of severe airflow obstruction, NYHA class III-IV, and duration of follow-up, and this may explain the inconsistent results obtained as regards the prognostic impact of COPD in HF.

Recently, Miniati et al. reported that a reduction of FEV_1_ – be it due to airflow obstruction or ventilatory restriction – is a significant, independent predictor of reduced survival in 439 patients with systolic HF [14]. In that study, the adjusted HR of death was 1.8-fold higher in patients with FEV_1_ <80% predicted as compared with those having FEV_1_ ≥80% [14].

As of now, limited data is available on the prognostic relevance of lung function tests other than spirometry. It is well established that chronic HF brings about extensive remodeling of the pulmonary intra-alveolar vessels and interstitium [26], which may hamper the exchange of gases across the alveolar-capillary barrier [15]. In 106 patients with systolic HF, Guazzi and coworkers reported that alveolar-capillary membrane conductance of less than 24.7 mL/min/mmHg is significantly and independently associated with a worse outcome [16]. Partitioning of lung diffusing capacity into its membrane and capillary blood volume components requires the determination of DL_CO_ at two different concentrations of oxygen in the inspired test bolus [15,16]. This technique is seldom used in routine clinical practice.

V_A_ represents an estimate of the lung gas volume into which CO is distributed and, therefore, is critical in the measurement of DL_CO_ with the single-breath approach [17]. In the presence of uneven distribution of ventilation, due to altered lung distensibility or airflow obstruction, V_A_ primarily reflects the volume of the airspaces into which the tracer gas rapidly equilibrates during the breath-hold time [17]. The estimated volume can, therefore, be regarded as reflecting the size of the well-ventilated lung regions. As shown in Table 2, spirometrically determined ventilatory restriction, was present in only 53 (39%) of 135 patients with V_A_ <80% predicted. So, we believe that measuring V_A_ may add valuable information as regards lung function assessment in patients with systolic HF.

Undoubtedly, factors other than V_A_ affect the lung diffusing capacity, including the physical properties of the alveolar membrane, the density of alveolar capillaries, and the hemoglobin concentration [17]. Thus, in multivariate analysis, we incorporated DL_CO_ among the potential confounding variables. After adjustment, the HR of death associated with the lower V_A_ category remained statistically significant.

### Study limitations

First, we only studied patients with systolic HF, so our findings may not apply to patients with isolated diastolic heart dysfunction. Second, our data originated from a single referral center, and included a relatively small number of cases. Broader multicenter prospective studies are needed to firmly establish the prognostic value of V_A_ in HF. Third, we obtained a single determination of V_A_. So, it would of interest to evaluate the effect of cardiovascular and pulmonary medications on V_A_ size over time, and to test whether improvement in V_A_ is associated with a better oucome in patients with chronic HF.

## Conclusions

The present study indicates that V_A_, as percent of the predicted value, is a significant, independent predictor of reduced survival in patients with systolic HF.

## Competing interests

The authors declare that they have no competing interests.

## Authors’ contribution

Study design (MM). Data collection (CP, ME, RP, IP). Data analysis (MM, SM, MB). Drafting the manuscript (MM). Approval of the final version of the manuscript (all authors).

## Pre-publication history

The pre-publication history for this paper can be accessed here:

http://www.biomedcentral.com/1471-2466/13/69/prepub
